# Effect of remimazolam versus sevoflurane on intraoperative hemodynamics in noncardiac surgery: a retrospective observational study using propensity score matching

**DOI:** 10.1186/s40981-023-00661-5

**Published:** 2023-10-26

**Authors:** Takayuki Katsuragawa, Soichiro Mimuro, Tsunehisa Sato, Yoshitaka Aoki, Matsuyuki Doi, Takasumi Katoh, Yoshiki Nakajima

**Affiliations:** Department of Anesthesiology and Intensive Care Medicine, Hamamatsu University School of Medicine, 1-20-1 Handayama, Higashi-Ku, Hamamatsu, Shizuoka, 431-3192 Japan

**Keywords:** Remimazolam, Intraoperative hypotension, Noncardiac surgery, Propensity score matching

## Abstract

**Background:**

This study compared the effects of remimazolam and sevoflurane on intraoperative hemodynamics including intraoperative hypotension (IOH).

**Results:**

This study involved adult patients undergoing noncardiac surgery using remimazolam (Group R) or sevoflurane (Group S) for maintenance anesthesia, and invasive arterial pressure measurements, from September 2020 to March 2023 at our hospital. IOH was defined as a mean blood pressure < 65 mmHg occurring for a cumulative duration of at least 10 min. A 1:1 propensity score-matching method was used. The primary endpoint was the occurrence of IOH, and the secondary endpoints were the cumulative hypotensive time, incidence of vasopressor use, and dose of vasopressor used (ephedrine, phenylephrine, dopamine, and noradrenaline). Group R comprised 169 patients, Group S comprised 393 patients, and a matched cohort of 141 patients was created by propensity score matching. There was no significant difference in the incidence of IOH between the two groups (85.1% in Group R vs. 91.5% in Group S, *p* = 0.138). Patients in Group R had a significantly lower cumulative hypotension duration (55 [18–119] vs. 83 [39–144] min, *p* = 0.005), vasopressor use (81.6% vs. 91.5%, *p* = 0.023), and dose of ephedrine (4 [0–8] vs. 12 [4–20] mg, *p* < 0.001) than those in Group S. There were no significant differences in the doses of other vasopressors between groups.

**Conclusions:**

Compared with sevoflurane, the maintenance of anesthesia with remimazolam was not associated with a decreased incidence of IOH; however, it reduced the cumulative hypotension time, incidence of vasopressor use, and dose of ephedrine.

**Supplementary Information:**

The online version contains supplementary material available at 10.1186/s40981-023-00661-5.

## Background

Intraoperative hypotension (IOH) is a common complication in noncardiac surgery. Numerous definitions of IOH have been used in previous studies, and the reported incidence rate has ranged from 5 to 99% [1, 2]. IOH is associated with reduced organ perfusion and negative outcomes for the kidneys, heart, and nervous system [3–5]. The severity and duration of hypotension have been shown to be associated with the incidence of adverse events [6, 7].

Remimazolam is used as a sedative for the induction and maintenance of general anesthesia [8]. Anesthesia with remimazolam has the advantage of hemodynamic stability and has been shown to reduce the risk of hypotension compared with the induction of anesthesia using propofol [9]. Moreover, compared with sevoflurane, remimazolam reduced the use of vasopressors and maintained a higher mean arterial pressure [10, 11]. However, the incidence of IOH has not been studied, and no studies have compared the effects of anesthetic maintenance with remimazolam versus sevoflurane on reducing the frequency of IOH.

In this study, we compared the frequency of IOH during noncardiac surgery in which anesthesia was maintained with remimazolam or sevoflurane.

## Methods

This single-institution retrospective study was approved by the Ethics Committee of Hamamatsu University Hospital, Hamamatsu, Japan (approval number 23–062). Because it was a retrospective study, the requirement to obtain informed consent was waived by the Ethics Committee.

### Patient selection

Patients who underwent noncardiac surgery under general anesthesia at our institution from September 2020 to March 2023 were retrospectively studied. Patients aged ≥ 20 years who underwent invasive arterial pressure measurements and in whom only remimazolam or sevoflurane was used for anesthetic maintenance were included. Patients who discontinued surgery, had a < 10-min observation period for arterial pressure measurement, or had incomplete data were excluded. The patients were divided into two groups according to the drug used for anesthetic maintenance: the sevoflurane group (Group S) and remimazolam group (Group R). The choice of anesthetic drug, intraoperative anesthetic depth, and hemodynamic control were at the discretion of the anesthesiologist in charge of the patient. Electroencephalography monitoring was used for patients with SedLine® (Masimo Corporation, Irvine, CA, USA) or Bispectral Index™ monitors (Medtronic Inc., Minneapolis, MN, USA) as needed.

### Perioperative variables

Baseline characteristics including age, sex, body mass index (BMI), American Society of Anesthesiologists physical status (ASA-PS), comorbidities, and medication history were recorded for each patient. In addition, the surgical time, infusion volume, urine output, blood loss, anesthetic use (the mean infusion rate of remifentanil and remimazolam and the mean sevoflurane concentration), use of any vasopressors (ephedrine, phenylephrine, dopamine, or noradrenaline), and doses of vasopressors were investigated.

### Intraoperative blood pressure

A catheter was inserted into the radial artery and the mean blood pressure was evaluated from the start to the end of the observation period for arterial pressure measurements. Invasive arterial pressure was recorded at 1-min intervals. Artifacts were removed using the following criteria: (1) out-of-range blood pressure as defined by (a) systolic blood pressure (SBP) ≥ 300 or ≤ 20 mmHg, (b) SBP ≤ diastolic blood pressure (DBP) + 5 mmHg, or (c) DBP ≤ 5 or ≥ 225 mmHg, and (d) mean arterial pressure ≤ 25 mmHg; and (2) abrupt changes in SBP ≥ 80 mmHg within 1 min in either direction or ≥ 40 mmHg within 2 min in both directions. Blood pressures between measurements were interpolated linearly.

The hypotensive time was recorded as the time for which the mean arterial pressure was < 65 mmHg and the IOH was defined as a cumulative hypotensive time of ≥ 10 min [7, 12]. The primary outcome was the difference in the incidence of IOH between the two groups. The secondary outcomes were the cumulative hypotensive time, incidence of vasopressor use, and intraoperative dose of vasopressors used (ephedrine, phenylephrine, dopamine, and noradrenaline).

### Statistical analysis

Continuous variables are presented as the mean (standard deviation) or median (interquartile range), and categorical variables are presented as a number (percentage).

Continuous variables were tested for normality with the Shapiro–Wilk test. Comparisons between groups were performed with a *t-*test for data following a normal distribution, and asymmetric data were analyzed using the Mann–Whitney *U*-test. Categorical variables were compared with the chi-square test or Fisher’s direct probability test, as appropriate.

Propensity score matching was performed to match the study groups using logistic regression analysis including the following potential confounding factors as independent variables: age, sex, BMI, ASA-PS, diabetes, hypertension, coronary artery disease, cerebrovascular disease, angiotensin-converting enzyme inhibitors or angiotensin II receptor blockers, concurrent epidural anesthesia, scheduled or emergency surgery, surgical site, and scheduled operative time. The nearest-neighbor matching method (1:1 ratio) was applied, with a caliper width of 0.2 for the logit-transformed propensity score. Variables in a matched data set were considered balanced between the groups if the standardized mean difference was < 0.1. All statistical analyses were performed using R software version 4.3.0 (R Development Core Team, Vienna, Austria). The R package “Matching” was used for the propensity score matching. All *p*-values < 0.05 were considered statistically significant.

## Results

**Fig. 1:**
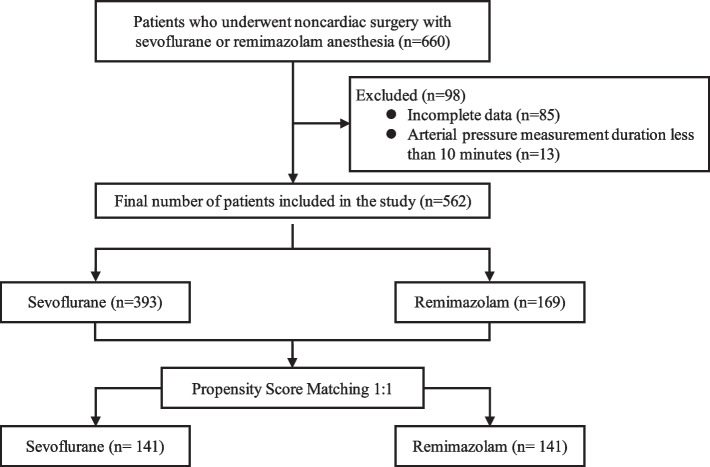
Flow diagram of the study

Overall, 660 patients were screened from September 2020 to March 2023. Of these patients, 85 were excluded because of missing BMI data and 13 were excluded because of a < 10-min observation period for arterial pressure measurement. Thus, 562 patients were included in the final analysis. After propensity score matching, 141 patients in each group were included (Fig. 1). Before propensity score matching, the parameters of age, sex, ASA-PS, coronary artery disease, type of surgery, combined with epidural anesthesia, and scheduled surgery were significantly different between the two groups (Table 1). After propensity score matching, there were no significant differences in any of the covariates, and the two groups were almost balanced (Table 1). The intraoperative parameters are shown in Table 2. The dose of remifentanil was significantly higher in Group R than in Group S. There were no significant differences in the operative time, blood loss, or infusion volume between the two groups; additionally, the frequency of IOH was not significantly different between the two groups (85.1% in Group R vs. 91.5% in Group S, *p* = 0.138). The cumulative hypotension time was significantly lower in Group R than in Group S (55 [18–119] vs. 83 [39–144] min, *p* = 0.005) and Group R also had a significantly lower incidence of vasopressor use (81.6% vs. 91.5%, *p* = 0.023) and ephedrine dose (4 [0–8] vs. 12 [4–20] mg, *p* < 0.001) than Group S. No significant differences were found for other vasopressors. There was no difference in mean blood pressure at the start of surgery, but the mean blood pressure at the end of surgery was higher in Group R (67 [59–74] vs. 64 [56–73] mmHg, *p* = 0.312 and 69 [62–75] vs. 63 [58–72] mmHg, *p* = 0.001) compared with Group S. The heart rate was higher in Group R at the start and at the end of surgery (66 [60–76] vs. 62 [55–69] mmHg, *p* = 0.003 and 70 [63–81] vs. 67 [60–75] mmHg, *p* = 0.006) (Supplementary Table 1) compared with Group S.

**Table 1:** Patient characteristics before and after propensity score matching

	Total cohort				Propensity score-matched cohort			
	Group S	Group R	P	SMD	Group S	Group R	P	SMD
	(*n* = 393)	(*n* = 169)			(*n* = 141)	(*n* = 141)		
Age (year)	69.0 (58.0–76.0)	73.0 (65.0–81.0)	< 0.001	0.388	71.0 (62.0–78.0)	72.0 (64.0–78.0)	0.392	0.085
Male	252 (64.1%)	87 (51.5%)	0.007	0.258	70 (49.6%)	73 (51.8%)	0.812	0.043
BMI (kg/m2)	22.1 (19.9–24.6)	22.0 (19.4–24.7)	0.592	0.041	22.2 (19.7–24.6)	22.0 (19.4–24.7)	0.894	0.036
ASA-PS			< 0.001	0.459			0.742	0.133
Class 1	23 (5.9%)							
Class 2	291 (74.0%)	7 (4.1%)			4 (2.8%)	7 (5.0%)		
Class 3	78 (19.8%)	94 (55.6%)			88 (62.4%)	84 (59.6%)		
Class 4	1 (0.3%)	65 (38.5%)			48 (34.0%)	48 (34.0%)		
Comorbidities		3 (1.8%)			1 (0.7%)	2 (1.4%)		
Hypertension	136 (34.6%)							
CAD	28 (7.1%)	73 (43.2%)	0.066	0.177	56 (39.7%)	57 (40.4%)	1	0.014
CVD	33 (8.4%)	22 (13.0%)	0.037	0.197	16 (11.3%)	14 (9.9%)	0.847	0.046
Diabetes mellitus	55 (14.0%)	13 (7.7%)	0.911	0.026	10 (7.1%)	10 (7.1%)	1	< 0.001
Medication history		27 (16.0%)	0.631	0.056	24 (17.0%)	23 (16.3%)	1	0.019
ACEI or ARB	91 (23.2%)							
Operative profiles		45 (26.6%)	0.439	0.08	36 (25.5%)	36 (25.5%)	1	< 0.001
Type of surgery								
General surgery	97 (24.7%)	57 (33.7%)	< 0.001	0.732	45 (31.9%)	49 (34.8%)	0.957	0.171
Gynecology Surgery	16 (4.1%)	6 (3.6%)			5 (3.5%)	6 (4.3%)		
Hepatobiliary Surgery	24 (6.1%)	3 (1.8%)			5 (3.5%)	3 (2.1%)		
Neurosurgery	37 (9.4%)	15 (8.9%)			17 (12.1%)	15 (10.6%)		
Orthopedic surgery	25 (6.4%)	38 (22.5%)			23 (16.3%)	23 (16.3%)		
Other surgery	84 (21.4%)	8 (4.7%)			12 (8.5%)	8 (5.7%)		
Thoracic surgery	85 (21.6%)	33 (19.5%)			24 (17.0%)	28 (19.9%)		
Urological surgery	25 (6.4%)	9 (5.3%)			10 (7.1%)	9 (6.4%)		
Combined with epidural anesthesia	141 (35.9%)	43 (25.4%)	0.02	0.228	34 (24.1%)	42 (29.8%)	0.347	0.128
Scheduled surgery	375 (95.4%)	151 (89.3%)	0.012	0.23	128 (90.8%)	131 (92.9%)	0.663	0.078

Data are expressed as the median (interquartile range) or number (%) of patients

*BMI* body mass index, *ASA-PS* American Society of Anesthesiologists physical status, *CAD* coronary artery disease, *CVD* cerebrovascular disease, *ACEI* angiotensin-converting enzyme inhibitors, *ARB* angiotensin II receptor blockers, *SMD* standardized mean difference

**Table 2:** Intraoperative variables before and after propensity score matching

	Total cohort			Propensity score-matched cohort		
	Group S	Group R	P	Group S	Group R	P
	(*n* = 393)	(*n* = 169)		(*n* = 141)	(*n* = 141)	
Intraoperative variables						
Operation time (min)	335 (166–498)	159 (100–261)	< 0.001	166 (112–290)	183 (111–293)	0.99
Anesthesia time (min)	417 (241–578)	241 (174–356)	< 0.001	240 (189–370)	257 (180–381)	0.86
Fluid intake (mL)	2750 (1500–4100)	1640 (1110–2450)	< 0.001	1550 (1100–2400)	1670 (1150–2600)	0.39
Blood loss (mL)	120 (24–360)	50 (10–190)	0	46 (10–156)	60 (10–185)	0.38
Urine output (mL)	325 (135–615)	130 (50–270)	< 0.001	200 (65–401)	155 (55–300)	0.2
Sevoflurane (%)	1.4 (1.2–1.5)			1.3 (1.1–1.5)		
Remimazolam (mg/kg/h)		0.72 (0.52–0.89)			0.76 (0.56–0.89)	
Remifentanil (μg/kg/min)	0.15 (0.11–0.20)	0.17 (0.12–0.21)	0.04	0.15 (0.11–0.19)	0.17 (0.12–0.22)	0
Fentanyl (mg)	0.15 (0–0.40)	0.20 (0.10–0.35)	0.15	0.20 (0.10–0.32)	0.20 (0.10–0.35)	0.44
Use of vasopressor	357 (90.8%)	137 (81.1%)	0	129 (91.5%)	115 (81.6%)	0.02
Ephedrine (mg)	10 (4–18)	4 (0–8)	< 0.001	12 (4–20)	4 (0–8)	< 0.001
Phenylephrine (mg)	0.10 (0–0.50)	0.05 (0–0.30)	0.06	0.10 (0–0.50)	0.05 (0–0.25)	0.06
Dopamine (mg)	0 (0–0)	0 (0–0)	0.3	0 (0–0)	0 (0–0)	0.45
Norepinephrine (mg)	0 (0–0)	0 (0–0)	0.17	0 (0–0)	0 (0–0)	0.56
Incidence of IOH	363 (92.4%)	144 (85.2%)	0.01	129 (91.5%)	120 (85.1%)	0.14
Cumulative hypotension time (min)	335 (166–498)	159 (100–261)	< 0.001	83 (39–144)	55 (18–119)	0.01

Data are expressed as the median (interquartile range) or number (%) of patients

*IOH* intraoperative hypotension, *SMD* standardized mean difference

## Discussion

To the best of our knowledge, this is the first study to compare the incidence of IOH among patients using remimazolam versus sevoflurane for anesthetic maintenance in noncardiac surgery. Patients who underwent anesthetic maintenance with remimazolam did not have a lower incidence of IOH than patients who underwent maintenance with sevoflurane, but they did have a decreased cumulative hypotensive time. In addition, the incidence of vasopressor use and the dose of ephedrine were reduced.

The definition of IOH varies from study to study. An intraoperative mean arterial pressure < 60–70 mmHg was associated with myocardial injury, acute kidney injury, and increased mortality, and organ damage was influenced by the severity and duration of hypotension [7, 13]. In previous reports, the incidence of IOH was 31.3% to 49.7% when defined as the occurrence of hypotension for at least 10 min with a mean arterial pressure threshold of 65 mmHg [1, 14, 15]. The overall incidence of IOH in the present study was 88.3%, which is higher than that in previous reports. This may have occurred because our study included patients who required invasive arterial pressure monitoring; thus, many critically ill patients or patients undergoing highly invasive surgeries were likely to be included in our study. In addition, hemodynamic management performed by the anesthesiologist in charge may have been affected.

In a previous study, anesthetic induction and maintenance with remimazolam in patients with severe aortic stenosis resulted in the reduced use of vasopressors compared with conventional anesthetics [10]. In another study, remimazolam was associated with maintenance of a higher mean blood pressure and reduced vasopressor use compared with sevoflurane in patients undergoing robotic gastrectomy [11]. The results of this study showed that the maintenance of anesthesia with remimazolam reduced the frequency of vasopressor use in noncardiac surgery, consistent with previous reports. In addition, despite no significant difference in the incidence of IOH, the cumulative hypotension time was reduced and the mean arterial pressure was maintained in Group R compared with Group S. These between-group differences occurred despite the administration of more ephedrine and less remifentanil in Group S. Furthermore, remimazolam was shown to cause less circulatory depression.

Intraoperative blood pressure elevation was suggested to be associated with postoperative complications, although not to the same extent as hypotension, and some reports indicated that intraoperative hypertension was a risk for postoperative acute kidney injury [13, 16]. In addition, there have been case reports of unexpected hypertension during the induction of anesthesia with remimazolam [17]. There was no significant difference in the frequency and duration of intraoperative hypertension in Group R compared with Group S (Supplementary Table 2). Remimazolam maintains stable hemodynamics and is useful for anesthesia, even in elderly patients and those with unstable cardiac dynamics.

The use of electroencephalogram (EEG) monitoring in this study was at the discretion of the anesthesiologist in charge. Therefore, it was difficult to compare the depth of anesthesia between the two groups because EEG monitoring was performed in a limited number of cases and only in a few cases in Group S (Supplementary Table 3). The heart rate at the start and end of surgery was significantly higher in Group R compared with Group S. Previous studies have reported that single-agent remimazolam increased the heart rate [18]. Furthermore, the maintenance of anesthesia with remimazolam resulted in a significantly higher heart rate compared with sevoflurane and propofol [11, 10]. However, a higher heart rate did not seem to indicate that Group R had a shallower depth of anesthesia compared with Group S.

This study had some limitations. First, it was a single‐center study; thus, the findings may not have high generalizability. Second, this was a retrospective study, the anesthetic management may have been performed at the discretion of the anesthesiologist in charge, and the anesthetic depth may have differed between the groups. Prospective studies are needed to clarify the effects of remimazolam. Third, only the invasive arterial pressure was recorded. It is possible that hypotension before placement of the arterial line was overlooked because propofol was used during the induction of anesthesia in many cases in Group S (Supplementary Table 4). However, in previous reports, post-induction hypotension was not associated with organ damage and did not appear to be as important as hypotension during surgery [19].

## Conclusions

Patients in whom remimazolam was used for the maintenance of general anesthesia were not associated with a decreased incidence of IOH compared with patients in whom sevoflurane was used; however, they had a decreased incidence of vasopressor use, dose of ephedrine, and cumulative hypotensive time. Remimazolam appears to maintain stable hemodynamics during anesthetic management in noncardiac surgery.

### Supplementary Information

**Additional file 1: Supplementary Table 1.** Intraoperative heart rate and mean blood pressure.

**Additional file 2: Supplementary Table 2.** Intraoperative hypertension.

**Additional file 3: Supplementary Table 3.** Number of cases using intraoperative EEG monitoring.

**Additional file 4: Supplementary Table 4.** Sedative and arterial line during the induction of anesthesia.

## Data Availability

The datasets used and/or analyzed during the current study are available from the corresponding author upon reasonable request.

## Abbreviations

IOH: Intraoperative hypotension
BMI: Body mass index
ASA-PS: American Society of Anesthesiologists physical status
SBP: Systolic blood pressure
DBP: Diastolic blood pressure
EEG: Electroencephalogram
